# Integration of inspiratory and expiratory intra-abdominal pressure: a novel concept looking at mean intra-abdominal pressure

**DOI:** 10.1186/2110-5820-2-S1-S18

**Published:** 2012-12-20

**Authors:** Siavash Ahmadi-Noorbakhsh, Manu LNG Malbrain

**Affiliations:** 1 Saadat Abad Veterinary Specialty Clinic, Saadat Abad, Tehran, Iran; 2 Executive Committee, World Society of the Abdominal Compartment Syndrome (WSACS), Dreef 1, Lovenjoel, 3360, Belgium; 3 ICU and High Care Burn Unit, Department of Intensive Care, Ziekenhuis Netwerk Antwerpen (ZNA) Stuivenberg, Lange Beeldekensstraat 267, Antwerp, 2060, Belgium

## Abstract

**Background:**

The intra-abdominal pressure (IAP) is an important clinical parameter that can significantly change during respiration. Currently, IAP is recorded at end-expiration (IAP_ee_), while continuous IAP changes during respiration (ΔIAP) are ignored. Herein, a novel concept of considering continuous IAP changes during respiration is presented.

**Methods:**

Based on the geometric mean of the IAP waveform (MIAP), a mathematical model was developed for calculating respiratory-integrated MIAP (i.e. $\text{MIA}{\text{P}}_{\text{ri}}=\text{IA}{\text{P}}_{\text{ee}}+i\cdot \Delta \text{IAP}$), where '*i*' is the decimal fraction of the inspiratory time, and where ΔIAP can be calculated as the difference between the IAP at end-inspiration (IAP_ei_) minus IAP_ee_. The effect of various parameters on IAP_ee _and MIAP_ri _was evaluated with a mathematical model and validated afterwards in six mechanically ventilated patients. The MIAP of the patients was also calculated using a CiMON monitor (Pulsion Medical Systems, Munich, Germany). Several other parameters were recorded and used for comparison.

**Results:**

The human study confirmed the mathematical modelling, showing that MIAP_ri _correlates well with MIAP (*R*^2 ^= 0.99); MIAP_ri _was significantly higher than IAP_ee _under all conditions that were used to examine the effects of changes in IAP_ee_, the inspiratory/expiratory (*I*:*E*) ratio, and ΔIAP (*P <*0.001). Univariate Pearson regression analysis showed significant correlations between MIAP_ri _and IAP_ei _(*R *= 0.99), IAP_ee _(*R *= 0.99), and ΔIAP (*R *= 0.78) (*P <*0.001); multivariate regression analysis confirmed that IAP_ee _(mainly affected by the level of positive end-expiratory pressure, PEEP), ΔIAP, and the *I*:*E *ratio are independent variables (*P <*0.001) determining MIAP. According to the results of a regression analysis, MIAP can also be calculated as

$$\text{MIAP}=-0.\text{3}+\text{IA}{\text{P}}_{\text{ee}}+0.\text{4}\cdot \Delta \text{IAP}+0.\text{5}\cdot \frac{I}{E}.$$

**Conclusions:**

We believe that the novel concept of MIAP is a better representation of IAP (especially in mechanically ventilated patients) because MIAP takes into account the IAP changes during respiration. The MIAP can be estimated by the MIAP_ri _equation. Since MIAP_ri _is almost always greater than the classic IAP, this may have implications on end-organ function during intra-abdominal hypertension. Further clinical studies are necessary to evaluate the physiological effects of MIAP.

## Introduction

The intra-abdominal pressure (IAP) is an important clinical parameter with major prognostic impact [1,2]. An unrecognised pathological increase in IAP eventually leads to intra-abdominal hypertension (IAH) and abdominal compartment syndrome (ACS) [3,4], which result in significant morbidity and mortality [5]. Thus, recognition and reliable measurement of IAP are the first important steps for prevention and management of IAH and ACS in critically ill patients [6].

**Figure 1:**
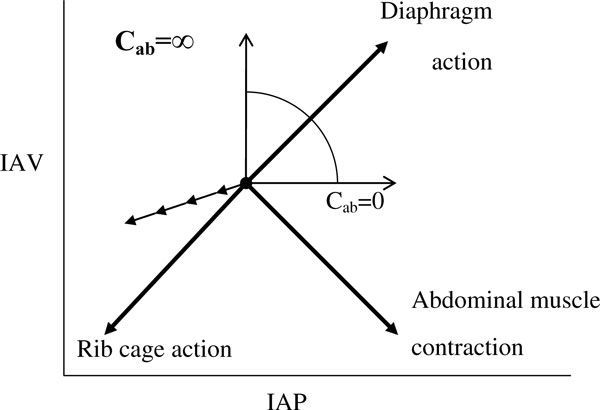
**Relationship between intra-abdominal volume (IAV), abdominal wall compliance (*C*_ab_) and intra-abdominal pressure (IAP)**. The directions of the movement of IAP on the *x *axis and IAV on the *y *axis associated with the isolated action of the rib cage inspiratory muscles, abdominal expiratory muscles, and the diaphragm are shown. The direction of the action of the diaphragm depends on the abdominal compliance. Adapted from de Keulenaer et al. [7].

**Figure 2:**
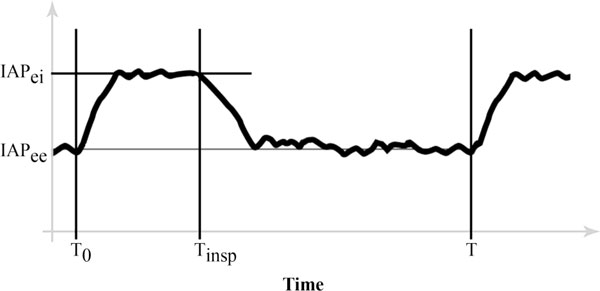
**Effect of respiration on intra-abdominal pressure (IAP)**. *T*_0_, start of inspiration; *T*_insp_, inspiratory time; *T*, total respiration time; IAP_ee_, end-expiratory IAP; IAP_ei_, end-inspiratory IAP.

Assuming no respiratory movement, the IAP would be relatively constant and primarily determined by body posture and anthropomorphy (e.g. body mass index) [3,7]. The IAP may be affected by conditions influencing intra-abdominal volume and abdominal compliance (*C*_ab_) [3,8,9]. Further, the complex interaction between intra-abdominal volume and *C*_ab _during respiration (Figure 1) may significantly [10] and frequently (12 to 40 changes per minute) change the IAP (Figure 2), with more intense effects during positive-pressure mechanical ventilation or the presence of positive end-expiratory pressure (PEEP) [10-12].

According to the current consensus definitions of the World Society of the Abdominal Compartment Syndrome (WSACS), the IAP should be measured at end-expiration (IAP_ee_) [13], referred to as the 'classic IAP' throughout the text. However, the IAP_ee _is only a single component of an ever-changing trend and thus does not incorporate a considerable portion of this IAP trend (Figure 2). The objectives of this study were to develop and validate a novel IAP measurement concept to consider IAP changes during respiration and to identify independent variables influencing IAP within this novel concept.

## Methods

### Part A: mathematical model

A set of numerous IAP values occurs for a patient during a single respiratory cycle. The central tendency of a set of values can be calculated by the mathematical function of the 'mean'. In determining the mean IAP, the arithmetic mean for IAP_ee _and the end-inspiratory IAP (IAP_ei_) was described previously [14], calculated by dividing the sum of the values by the number of values. However, employing the arithmetic mean for the IAP waveform is mathematically incorrect. Instead, the mean of a waveform can be calculated by the 'geometric mean' function. The geometric mean is calculated by dividing the area under the waveform in a definite interval (i.e. the definite integral of the waveform) by the value of the definite interval [15]. Therefore, the mean IAP (MIAP) for a sample IAP waveform between the times (*T*_0_) and (*T*) in Figure 2 can be calculated as follows:

(1) $$\text{MIA}{\text{P}}_{\text{ri}}=\left(\frac{1}{T-T_{0}}\right)\cdot \overset{T}{\underset{T_{0}}{\int }}\text{IAP}\left(t\right)\text{ dt},$$

where 'MIAP_ri_' is the respiratory-integrated MIAP, '*T*−*T*_0_' is the time interval for a full respiratory cycle, and 'IAP (*t*) dt' is the IAP at each time point (*t*). The result would be a time-weighted mean for the IAP waveform. This is closely analogous with the critically important cardiovascular concept of mean arterial blood pressure [16-18], which is the geometric mean of the arterial blood pressure waveform [19,20]. Equation 1 may be simplified as follows (see the addendum)[21]:

(2) $$\text{MIA}{\text{P}}_{\text{ri}}=\text{IA}{\text{P}}_{\text{ee}}+i\cdot \Delta \text{IAP,}$$

where '*i*' is the decimal fraction of the inspiratory time in a respiratory cycle and can be calculated from the inspiratory/expiratory (*I*:*E*) ratio (*i *= *I */(*I *+ *E*); 0 <*i *< 1) and ΔIAP = IAP_ei _− IAP_ee_. Since IAP_ee_, *i*, and ΔIAP can be assumed to be independent, a computerised iteration can be used for a set of values for each parameter to determine their effect on MIAP_ri _and to compare the MIAP_ri _with the classic IAP.

**Figure 3:**
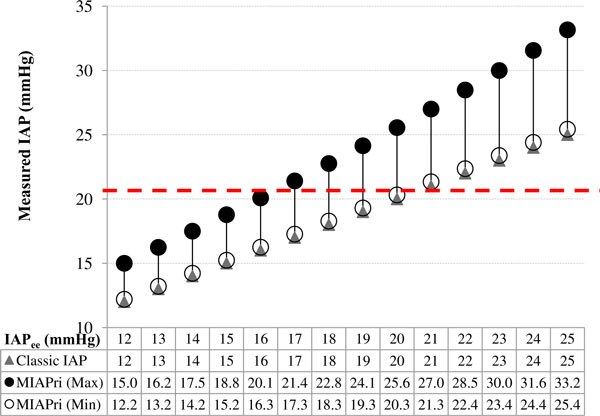
**Mathematical modelling of IAP measurements for various end-expiratory IAP values (IAP_ee_)**. The classic (IAP_ee_) and novel (MIAP_ri_) methods were used to measure the IAP. The dashed line represents the ACS threshold. The lines connecting the Max and Min MIAP_ri _values represent the range of possible MIAP_ri _values.

The effects of IAP_ee _on MIAP_ri _and the classic IAP were examined through a gradual increase of IAP_ee _from 12 to 25 mmHg, with steps of 1 mmHg (Figure 3). For each IAP_ee_, a range of possible MIAP_ri _values was calculated according to Equation 2 with an *I*:*E *ratio of 4:1 and an ΔIAP of 8.16 mmHg for the maximum MIAP_ri_, and an *I*:*E *ratio of 1:4 and an ΔIAP of 1 mmHg for the minimum MIAP_ri_. Because previous studies have shown a correlation between ΔIAP and IAP_ee_, the ΔIAP was increased 10% for each 1 mmHg increase in the IAP_ee_.

**Figure 4:**
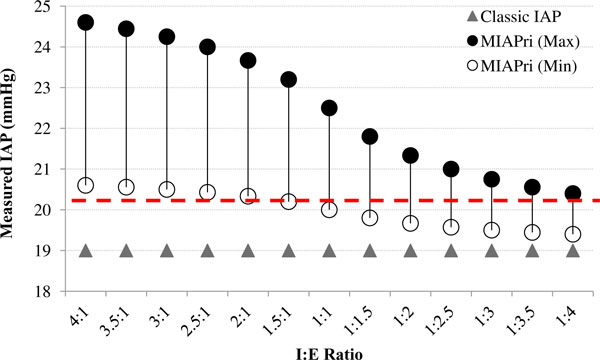
**Mathematical modelling of IAP measurements for a constant 19 mmHg end-expiratory IAP (IAP_ee_) and various *I*:*E *ratios**. The MIAP_ri _values were calculated for various *I*:*E *ratios. The classic (IAP_ee_) and novel (MIAP_ri_) methods were compared. For each *I*:*E *ratio, a range of possible MIAP_ri _values was calculated according to various ΔIAP values. The dashed line represents the ACS threshold.

The effects of the *I*:*E *ratio on MIAP_ri _and the classic IAP were examined by a gradual increase in the *I*:*E *ratio from 1:4 to 4:1 with steps of 0.5 (Figure 4). The amount of IAP_ee _was held constant (19 mmHg). For each *I*:*E *ratio, a range of possible MIAP_ri _values was calculated with an ΔIAP of 7 mmHg for the maximum MIAP_ri _and an ΔIAP of 2 mmHg for the minimum MIAP_ri_.

**Figure 5:**
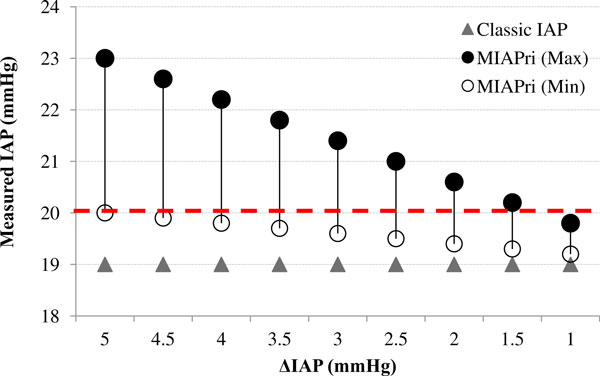
**Mathematical modelling of IAP measurement for a constant 19 mmHg end-expiratory IAP (IAP_ee_) and various ΔIAP**. The classic (IAP_ee_) and novel (MIAP_ri_) methods were used to measure the IAP. The MIAP_ri _values were calculated for each ΔIAP. A range of possible MIAP_ri _values for each ΔIAP was calculated according to various *I*:*E *ratios. The dashed line represents the ACS threshold.

The effects of ΔIAP on MIAP_ri _and the classic IAP were examined by a gradual increase in ΔIAP from 1 to 5 mmHg, with steps of 0.5 mmHg (Figure 5). The amount of IAP_ee _was held constant (19 mmHg). For each ΔIAP, a range of possible MIAP_ri _values was calculated with an *I*:*E *ratio of 4:1 for the maximum MIAP_ri _and an I:E ratio of 1:4 for the minimum MIAP_ri_.

Each of the abovementioned data sets was assumed to be a unique case, and the values shown in Figures 3,4,5 should not be considered as a trend in changes that can be obtained in a single patient.

### Part B: human pilot study

In six ICU patients that were mechanically ventilated with Evita XL ventilators (Draeger, Lubeck, Germany), the mean IAP was automatically calculated as the geometrical mean (MIAP) via a balloon-tipped nasogastric tube connected to a CiMON monitor (Pulsion Medical Systems, Munich, Germany). The MIAP_ri _was also calculated according to Equation 2. Data were collected on respiratory settings, plateau and mean alveolar pressures (*P*_plat_, *P*_mean_), PEEP, and dynamic compliance (calculated as the tidal volume (TV) divided by (*P*_plat _- PEEP)). The *C*_ab _was calculated as TV divided by ΔIAP. The thoraco-abdominal index of transmission (TAI) was calculated as Δ*P*_alv _(= *P*_plat _− PEEP) divided by ΔIAP, in which *P*_alv _is the alveolar pressure.

The effects of IAP_ee _on MIAP_ri _were examined by a gradual increase in PEEP from 0 to 15 cmH_2_O, with steps of 5 cmH_2_O during a best-PEEP manoeuvre (20 measurements at each PEEP level in five patients, resulting in 80 measurements). The effects of ΔIAP on MIAP_ri _were examined by a gradual increase in TV from 250 to 1,000 ml, with steps of 250 ml during a low-flow pressure-volume loop (20 measurements at each TV level in five patients, resulting in 80 measurements). The effects of *I*:*E *ratio on MIAP_ri _were examined by a gradual increase in the *I*:*E *ratio from 1:2 to 2:1, with steps of 0.5 during a recruitment manoeuvre (9 measurements at each *I*:*E *ratio in one patient, resulting in 45 measurements).

Statistical analysis was performed using SPSS software. Pearson correlation analysis and Bland and Altman analysis were performed. For comparisons between MIAP_ri _and IAP_ee _at different levels of IAP_ee _(PEEP), TV, and *I*:*E *ratio, a two-tailed paired Student's *t*-test was performed. Data are expressed as the mean with the standard deviation (SD), unless specified otherwise. A *P *value below 0.05 was considered statistically significant. The local EC and IRB approved the study, and informed consent was obtained from next of kin.

## Results

### Part A: mathematical modelling

According to Equation 2, three major independent parameters determine the MIAP_ri_: IAP_ee_, *I*:*E *ratio, and ΔIAP. Therefore, for each IAP_ee_, the MIAP_ri _depends on two other factors (Figure 3). For IAP_ee _values between 16 and 20 mmHg, the classic IAP remained below the ACS threshold (dashed line in Figure 3); however, the MIAP_ri _was able to exceed the ACS threshold. Furthermore, as seen in Figures 4 and 5, the classic IAP was continuously below the ACS threshold, but different ranges of probable MIAP_ri _values were above the ACS threshold. By changing the *I*:*E *ratio, the MIAP_ri _values changed with dissimilar intensities (e.g. when the *I*:*E *ratio decreased from 4:1 to 3.5:1, the intensity of changes in the MIAP_ri _values was less than that when the *I*:*E *ratio decreased from 1.5:1 to 1:1; Figure 4). Furthermore, for a constant IAP_ee_, higher values for either the *I*:*E *ratio or ΔIAP were found to be capable of causing a wider range of possible MIAP_ri _values (Figures 4 and 5). Mathematically, for all instances in which the ΔIAP was greater than 0 mmHg, the MIAP_ri _was larger than the classic IAP (see the addendum) [21].

**Figure 6:**
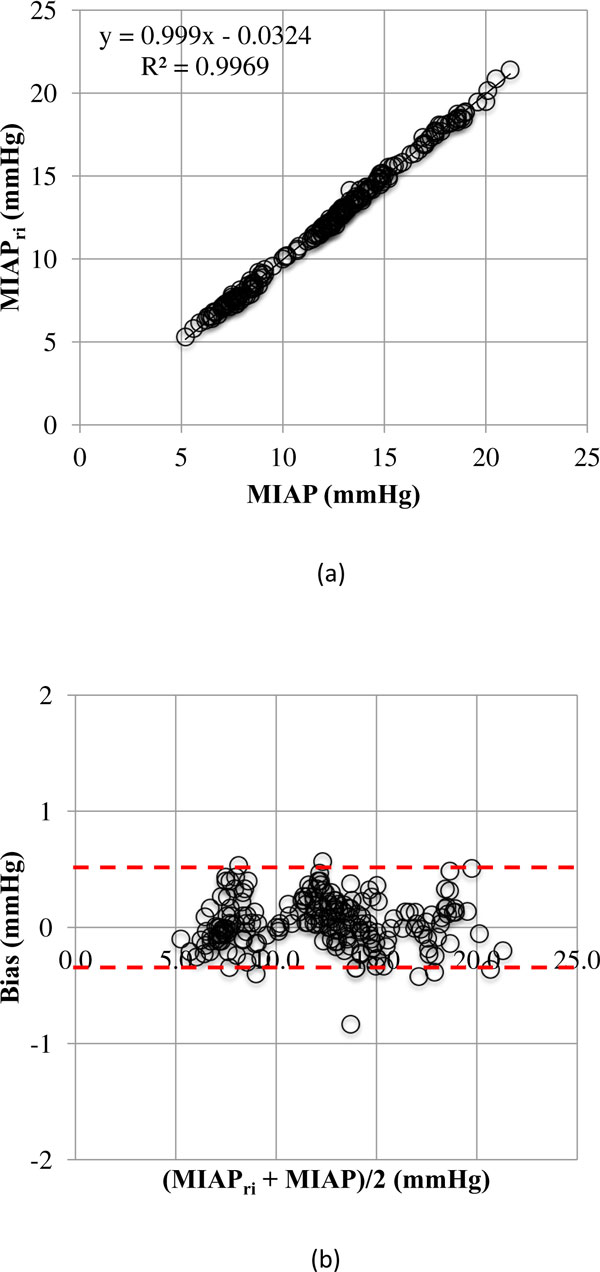
**Regression plot and Bland and Altman analysis**. (**A**) Regression plot comparing mean IAP measured via the geometric mean (MIAP) versus the respiratory-integrated MIAP (MIAP_ri_). There is an excellent correlation between the two methods. (**B**) Bland and Altman analysis comparing MIAP with MIAP_ri_. The dashed red lines show the upper and lower limits of agreements.

### Part B: human pilot study

Six mechanically ventilated patients (three severely burned patients and three surgical ICU patients) were studied. The male-to-female ratio was 2:1. Table 1 summarises the baseline patient demographics.

**Table 1:** Patient characteristics at baseline

Parameter	Mean ± SD
Age	59.5 ± 14.4
SAPS-II	43.5 ± 11.6
APACHE-II	21.8 ± 8.6
SOFA	9.5 ± 4
BMI (kg/m^2^)	28.6 ± 4.7
IAP_ei _(mmHg)	15.3 ± 3.7
IAP_ee _(mmHg)	11.1 ± 2.8
ΔIAP (mmHg)	4.3 ± 1.3
MIAP (mmHg)	12.9 ± 3
IBP (mmHg)	12 ± 3
TV (ml)	608 ± 117
TV (ml/kg)	7.2 ± 1.2
RR (/min)	17.7 ± 2.1
P_plat _(cmH_2_O)	28 ± 4.1
PEEP (cmH_2_O)	9.2 ± 3.3

SAPS, simplified acute physiology score; APACHE, acute physiology and chronic health evaluation; SOFA, sequential organ failure assessment; BMI, body mass index; IAP_ei_, end-inspiratory IAP; IAP_ee_, end-expiratory IAP; MIAP, mean IAP; IBP, intra-bladder pressure; TV, tidal volume; RR, respiratory rate; P_plat_, plateau airway pressure; PEEP, positive end-expiratory pressure.

#### Regression analysis and Bland and Altman analysis

In total, 205 paired MIAP and MIAP_ri _measurements were performed with an identical statistical mean of 12.2 ± 3.8 mmHg. Figure 6A shows an excellent correlation between the MIAP and MIAP_ri _(*R*^2 ^= 0.99, *P <*0.001). Analysis according to Bland and Altman showed a bias and precision of 0 and 0.2 mmHg, respectively, with small limits of agreement ranging from −0.4 to 0.5 mmHg (Figure 6B). The percentage error was 3.5%.

#### Effect of IAP_ee_, I:E ratio, and ΔIAP on MIAP_ri_

**Figure 7:**
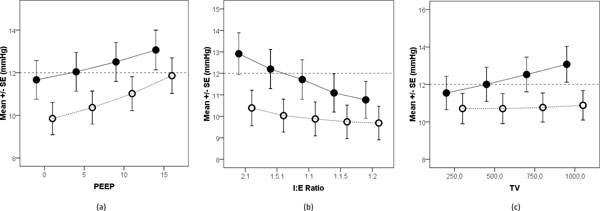
**The effects of gradual increase of PEEP, *I*:*E *ratio, and TV**. (**A**) The effect of gradual increase of PEEP on classic IAP (open circles) and the respiratory-integrated MIAP (MIAP_ri_; closed circles). Both the classic IAP and MIAP_ri _were increased significantly (*P <*0.001). The MIAP_ri _was significantly higher than the classic IAP for all PEEP levels (*P <*0.001). The dashed line shows the 12 mmHg IAH grade I threshold. (**B**) The effect of gradual increase of *I*:*E *ratio on IAP_ee _(open circles) and MIAP_ri _(closed circles). Both the IAP_ee _and MIAP_ri _were increased significantly (*P <*0.001). The MIAP_ri _was significantly higher than IAP_ee _for all *I*:*E *ratios (*P <*0.001). The dashed line represents the 12 mmHg IAH grade I threshold. (**C**) The effect of gradual increase of tidal volume (TV; and thus ΔIAP) on IAP_ee _(open circles) and MIAP_ri _(closed circles). The MIAP_ri _was significantly higher than IAP_ee _at all TV values (*P <*0.001). The dashed line shows the 12 mmHg IAH grade I threshold.

Gradually increasing PEEP from 0 to 15 cmH_2_O resulted in an increase in MIAP_ri _from 11.7 ± 4.1 to 13.1 ± 4.2 mmHg (*P <*0.001). Meanwhile, IAP_ee _increased from 9.9 ± 3.4 to 11.9 ± 3.7 mmHg (*P <*0.001). Moreover, a gradual increase in the *I*:*E *ratio from 0.5 (1:2) to 2 (2:1) caused an increase in MIAP_ri _from 10.8 ± 2.6 to 12.9 ± 2.9 mmHg (*P <*0.001), while IAP_ee _increased from 9.7 ± 2.3 to 10.4 ± 2.5 mmHg (*P <*0.001). In addition, gradually increasing TV from 250 to 1,000 ml led to an increase in ΔIAP from 2.1 ± 1.1 to 5.7 ± 2.3 (*P <*0.001). This increase in ΔIAP resulted in an increase in MIAP_ri _from 11.6 ± 4 to 13.1 ± 4.3 mmHg (*P <*0.001), while IAP_ee _increased from 10.7 ± 3.6 to 10.9 ± 3.5 mmHg (*P *= NS). The MIAP_ri _was significantly higher than IAP_ee _at each PEEP level, *I*:*E *ratio, and TV (Figure 7A,B,C; *P <*0.001).

The classic IAP of patients was below the IAH grade I threshold; however, the MIAP_ri _significantly exceeded the threshold in several instances (*P *< 0.001; Figure 7).

#### Univariate analysis

**Figure 8:**
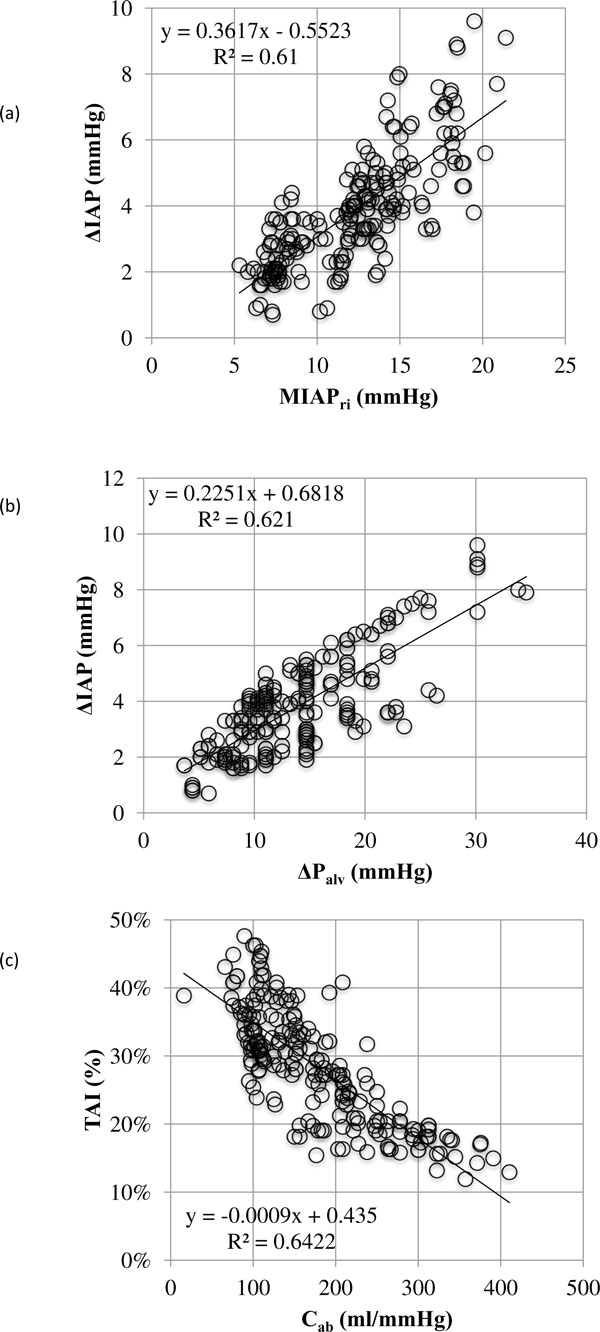
**Linear regression plots**. (**A**) Linear regression plot showing the respiratory-integrated mean intra-abdominal pressure (MIAP_ri_) in relation to ΔIAP (= IAP_ei _− IAP_ee_, where IAP_ei _is the end-inspiratory IAP and IAP_ee _is the end-expiratory IAP). (**B**) Linear regression plot showing the respiratory changes of intra-abdominal pressure (ΔIAP) in relation to Δ*P*_alv _(= P_plat _- PEEP, where *P*_alv _is the alveolar pressure, *P*_plat _is the plateau alveolar pressure, and PEEP is the positive end-expiratory pressure). (**C**) Linear regression plot showing the relation between the thoraco-abdominal index of transmission (i.e. TAI = Δ*P*_alv _/ΔIAP) and the abdominal wall compliance (i.e. *C*_ab _= TV/ΔIAP).

Univariate Pearson regression analysis showed significant correlations between MIAP_ri _and IAP_ei _(*R *= 0.99), IAP_ee _(*R *= 0.99), ΔIAP (*R *= 0.78), and *C*_ab _(*R *= −0.74); between IAP_ei _and IAP_ee _(*R *= 0.96), ΔIAP (*R *= 0.86), and *C*_ab _(*R *= −0.73); between IAP_ee _and ΔIAP (*R *= 0.7) and *C*_ab _(*R *= −0.73); between ΔIAP and Δ*P*_alv _(*R *= 0.79) and *C*_ab _(*R *= −0.58); and finally between TAI and *C*_ab _(*R *= −0.8) (*P <*0.001). Figure 8A,B,C shows some regression plots.

#### Multivariate regression analysis

Analyses showed that the IAP_ee _(mainly affected by PEEP), ΔIAP, and *I*:*E *ratio were independent variables defining the MIAP (Table 2). According to the regression analysis in our sample population, the MIAP can also be calculated from the following simplified formula (*P <*0.001), in which '*I*' and '*E*' are elements of the *I*:*E *ratio:

$$\text{MIA}{\text{P}}_{\text{ri}}=-0.3+\text{IA}{\text{P}}_{\text{ee}}+0.4\cdot \Delta \text{IAP + 0}\text{.5}\cdot \frac{I}{E}.$$

**Table 2:** Multiple regression analysis looking for independent variables influencing MIAP

	Unstandardized coefficients		Standardized coefficients			95.0% Confidence interval for *B*	
Model	*B*	Standard error	Beta	*t*	Significance	Lower bound	Upper bound
(Constant)	−0.27	0.20		−1.4	0.164	−0.66	0.11
PEEP	0.01	0.00	0.01	2.1	0.040	0.00	0.01
Δ*P*_alv_	0.01	0.01	0.02	1.5	0.133	0.00	0.02
*I*:*E *ratio	0.48	0.04	0.04	12.3	0.000	0.40	0.56
IAP_ee_	0.99	0.01	0.86	144.6	0.000	0.98	10.01
ΔIAP	0.35	0.03	0.16	11.3	0.000	0.29	0.41
TAI	0.00	0.00	0.01	1.1	0.259	0.00	0.01
*C*_ab_	0.00	0.00	−0.01	−1.6	0.105	0.00	0.00

PEEP, positive end-expiratory pressure; *P*_alv_, alveolar pressure; *I*:*E *ratio, inspiratory-to-expiratory ratio; IAP_ee_, end-expiratory IAP; TAI, thoraco-abdominal index of transmission; *C*_ab_, abdominal wall compliance.

## Discussion

A novel concept of IAP measurement based on the geometric mean of the IAP waveform was presented. The independent parameters determining the IAP in this concept were defined. The human pilot study validated the mathematical modelling with an excellent correlation. A significant difference was observed between the classic IAP and the MIAP_ri _in our clinical study.

The human study confirmed that MIAP_ri _is as accurate as an automated geometric MIAP calculation by a CiMON monitor. More importantly, the higher the MIAP or IAP_ee_, the higher the ΔIAP since ΔIAP acts as an indirect marker of *C*_ab_. The ΔIAP is correlated with Δ*P*_alv _or is thus inversely correlated with dynamic compliance. As well, the higher the *C*_ab_, the lower the TAI. The human study confirmed the predictions of the mathematical modelling in which IAP_ee _(affected by different PEEP settings), ΔIAP, and *I*:*E *ratio were recognised as the major independent determinants of MIAP_ri_. We also showed that in patients with IAH and under mechanical ventilation, the IAP may be influenced by ventilator settings.

The critical difference between the MIAP_ri _and the classic IAP near the ACS threshold in our mathematical modelling, as well as the significantly higher MIAP_ri _than the IAP_ee _around the IAH threshold in our human study, calls for future studies. The dissimilar intensity in MIAP_ri _changes under changes in the *I*:*E *ratio in Figure 4 may implicate the existence of critical points in the *I*:*E *ratio, wherein changing this ratio may cause a more intense change in the MIAP_ri_. Furthermore, since MIAP_ri _seems to be almost always larger than the classic IAP, relying only on the classic IAP may place some patients at risk of *silent *IAH or ACS. Although the aim of the current study was not to address these implications clinically, these findings indicate that further investigations should be performed on respiratory manoeuvres to manage IAH in mechanically ventilated patients (e.g. decreasing the *I*:*E *ratio and/or the ΔIAP, or maintaining the *I*:*E *ratio in a predefined range).

A limitation of our study was the lack of data to evaluate the physiological difference between the MIAP_ri _and the classic IAP. However, this study only aimed to prove the concept and to set the stage for further studies. Therefore, we believe that the lack of physiological data does not limit our findings. Nonetheless, further studies on the clinical effects of this concept are necessary before it can be introduced in clinical practice.

## Conclusions

A novel concept MIAP_ri _was presented to consider the IAP changes during respiration and was based on the geometric mean (MIAP) of the IAP waveform. An excellent correlation was observed between the results of the mathematical modelling and those obtained in real patients. Substantial differences were observed between the two IAP methods (the classic IAP measured at end expiration and the novel MIAP). Based on our findings, we believe that the novel concept of MIAP_ri _may be a better representation for the pressure concealed within the abdominal cavity. Further clinical studies are necessary to reveal the physiological effects of this novel concept.

## Abbreviations

ACS: abdominal compartment syndrome; *C*_ab_: abdominal compliance; IAH: intra-abdominal hypertension; IAP: intra-abdominal pressure; IAP_ee_: end-expiratory IAP; IAP_ei_: end-inspiratory IAP; MIAP: mean intra-abdominal pressure (geometrical mean); MIAP_ri_: respiratory-integrated mean intra-abdominal pressure; *P*_alv_: alveolar pressure; *P*_mean_: mean airway pressure; *P*_plat_: plateau airway pressure; PEEP: positive end-expiratory pressure; TAI: thoraco-abdominal index of transmission; TV: tidal volume; WSACS: World Society of the Abdominal Compartment Syndrome.

## Competing interests

MLNGM is a member of the medical advisory board of Pulsion Medical Systems, Munich, Germany.

## Authors' information

SA is aveterinary surgeon (DVM, DVSc) and a medical research consultant in laboratory animal researches in the field of trauma, haemorrhage, critical care, and anaesthesia. MLNGM is a former president and treasurer of the World Society of the Abdominal Compartment Syndrome and is the ICU and High Care Burn Unit Director of the Department of Intensive Care in Ziekenhuis Netwerk Antwerpen Stuivenberg.

## Authors' contributions

SA and MLNGM planned the study and were responsible for the design, coordination, and drafting the manuscript. SA developed the mathematical model for MIAP calculation and performed the theoretical analyses. MLNGM performed the data collection and statistical analysis for the human pilot study. Both authors read and approved the final manuscript.

## Addendum

See additional file 1.

## Supplementary Material

Additional file 1 **Mathematical model for calculation of mean intra-abdominal pressure, taking into account integration of inspiratory and expiratory intra-abdominal pressure**. Click here for file
