# Supplementary material for: Integration of inspiratory and expiratory intra-abdominal pressure: a novel concept looking at mean intra-abdominal pressure
Source: Ann Intensive Care. 2012 Dec 20;2(Suppl 1):S18. doi: 10.1186/2110-5820-2-S1-S18 (PMC3527153; doi:10.1186/2110-5820-2-S1-S18)
Supplement: Additional file 1 — Mathematical model for calculation of mean intra-abdominal pressure, taking into account integration of inspiratory and expiratory intra-abdominal pressure. [file 2110-5820-2-S1-S18-S1.docx]

**Addendum: Mathematical model for calculation of mean intra-abdominal pressure, taking into account integration of inspiratory and expiratory intra-abdominal pressure**

Siavash Ahmadi-Noorbakhsh^1^ and Manu L N G Malbrain^2^*

^1^ Saadat Abad Veterinary Specialty Clinic, Saadat Abad, Tehran, Iran

^2^ Department of Intensive Care, Ziekenhuis Netwerk Antwerpen (ZNA) Stuivenberg, Lange Beeldekensstraat 267, Antwerpen 6, 2060, Belgium

*Corresponding author: [manu.malbrain@skynet.be](mailto:manu.malbrain@skynet.be)

Email addresses:

SA: [s.noorb@gmail.com](mailto:s.noorb@gmail.com); [s.noorbakhsh@urmia.ac.ir](mailto:s.noorbakhsh@urmia.ac.ir);

MLNGM: [manu.malbrain@skynet.be](mailto:manu.malbrain@skynet.be)

In this issue of the journal, we presented a novel concept of intra-abdominal (IAP) measurement that accounts for continuous IAP changes during respiration [1]. This concept was based on the mathematical function of the ‘geometric mean’ for the IAP waveform and was termed the ‘respiratory integrated mean intra-abdominal pressure (MIAP_ri_)’. We presented some formulas to calculate the MIAP_ri_ and evaluated them in real patients. An excellent correlation was observed between the formulas and the results from the real patients. Although the mathematical calculations to achieve the formulas were important, we realised that their full development in the text would distract from the main scope because of their complexity. Herein, we would like to present a brief addendum on the mathematical calculations central to this paper.

According to the concept of geometric mean for a waveform graph [2,3], the mean of an IAP waveform is calculated by dividing the definite integral of the IAP waveform during one respiratory cycle by the time of the respiratory cycle. Therefore [1],

$MIAP_{ri}=\left(\frac{1}{T-T_{0}}\right)\cdot\int_{T_{0}}^{T} IAP(t)dt$ (1)

where the ‘*T*-*T*_0_’ is the respiratory time from the beginning of inspiration (*T*_0_) to the end of expiration (*T*); and ‘IAP (*t*) dt’ is the IAP value in each moment (*t*). Computers can easily calculate the integral of any IAP waveform; however in terms of manual calculations, presenting a universal formula for all of the various kinds of IAP waveform morphologies (ramp) is impossible. To solve the problem, Equation 1 can also be written as a limit of a Riemann sum, which is well described by Stewart [2];

$${MIAP}_{ri}=\lim_{n\to+\infty} \frac{1}{T-T_{0}}\cdot\sum_{t=1}^{n} IAP\left( t_{n} \right) \Delta t$$

where ‘*n*’ is the number of IAP measurements, IAP (*t_n_*) is the IAP in a moment ‘*t_n_*’, and Δ*t* is the time difference between two consecutive IAP measurements. The degree of accuracy of this Riemann sum is determined by the *n* number: the larger the *n*, the more accurate the result [2]. However, for clinical use one may only measure end-inspiratory IAP (IAP_ei_) and end-expiratory IAP (IAP_ee_). Thus, MIAP_ri_ would be approximately

$${MIAP}_{ri}=\lim_{n\to2} \frac{1}{T-T_{0}}\cdot\sum_{t=1}^{2} IAP\left( t_{n} \right) \cdot\Delta t=\frac{1}{T-T_{0}}\cdot\left[ IAP\left( t_{1} \right)\cdot\left( T_{insp}-T_{0} \right)+IAP\left( t_{2} \right) \cdot(T-T_{insp}) \right] (2)$$

where IAP (*t*_1_) stands for the IAP_ei_, IAP (*t*_2_) stands for the IAP_ee_, ‘*T*_insp_-*T*_0_’ is the inspiratory time, and the ‘*T*-*T*_insp_’ is the expiratory time. To simplify Equation 2, we can introduce variable ‘*i*’ as the decimal fraction of the inspiratory time in a respiratory cycle (0 < *i* <1). The *i* variable can be calculated from the I:E ratio as *i* = I/(I + E). Then

$${(T}_{insp}-T_{0})=i\cdot\left( T-T_{0} \right)$$

$(T-T_{insp})=\left( 1-i \right)\cdot(T-T_{0})$.

Therefore, Equation 2 can also be written as

$${MIAP}_{ri}=\frac{1}{T-T_{0}}\times\left[ {IAP}_{ei}\cdot i\cdot\left( T-T_{0} \right)+{IAP}_{ee}\cdot\left( 1-i \right)\cdot(T-T_{0}) \right]$$

Therefore,

$${MIAP}_{ri}={IAP}_{ei}\cdot i+{IAP}_{ee}\cdot\left( 1-i \right).$$

For ΔIAP = IAP_ei_ − IAP_ee_, then

${MIAP}_{ri}={IAP}_{ee}+i\cdot\Delta IAP$.

Therefore, the mathematical model suggests that the MIAP_ri_ depends on three major parameters: IAP_ee_, *i* and ΔIAP. In addition, the mathematical comparison between the MIAP_ri_ and the classic IAP is as follows:

$$\frac{{MIAP}_{ri}}{{IAP}_{ee}}=\left( \frac{\int_{T_{0}}^{T} IAP \left( t \right) dt}{\left( T-T_{0} \right)\cdot{IAP}_{ee}} \right)$$

which can also be approximated as follows, in which 0 < *i* < 1:

$$\frac{{MIAP}_{ri}}{{IAP}_{ee}}=1-i+i\cdot\left( \frac{{IAP}_{ei}}{{IAP}_{ee}} \right).$$

Therefore, for all ΔIAPs > 0 mmHg, the MIAP_ri_ is larger than the classic IAP.

**Abbreviations**

IAP, intra-abdominal pressure; IAP_ee_, end-expiratory IAP; IAP_ei_, end-inspiratory IAP; MIAP_ri_, respiratory integrated mean intra-abdominal pressure

**Competing interests**

MLNGM is member of the medical advisory board of Pulsion Medical Systems, Munich, Germany.

**Authors' information**

SA is a veterinary surgeon (DVM, DVSc) and a medical research consultant in laboratory animal researches in the field of trauma, haemorrhage, critical care, and anaesthesia. MLNGM is a former president and treasurer of the World Society of the Abdominal Compartment Syndrome and is the ICU and High Care Burn Unit Director of the Department of Intensive Care in Ziekenhuis Netwerk Antwerpen Stuivenberg.

**Authors' contributions**

SA and MM performed the literature review and were responsible for the design, coordination, and drafting the manuscript. SA developed the mathematical model for MIAP calculation and performed the theoretical analyses. Both authors read and approved the final manuscript.

**References**

1. Ahmadi-noorbakhsh S, Malbrain M: **Integration of the inspiratory and expiratory intra-abdominal pressure (IAP): a novel fundamental concept looking at mean intra-abdominal pressure (MIAP).** *Ann Intensive Care* 2012, **2**(Suppl 1):S18**.**

2. Stewart J: *Calculus: Concepts and Contexts.* 3rd edition. Edited by Belmont, CA: Brooks Cole: Belmont; 2005:104–110, 343–353, 460–463.

3. Gowers T, Barrow-Green J, Leader I: *The Princeton Companion to Mathematics.* Princeton University Press: Princeton; 2008:175–180, 703.
